# AI-Techniques Loss-Based Algorithm for Severity Classification (ATLAS): a novel approach for continuous quantification of exertional symptoms during incremental exercise testing

**DOI:** 10.1093/jamia/ocaf051

**Published:** 2025-03-27

**Authors:** Abed A Hijleh, Sophia Wang, Danilo C Berton, Igor Neder-Serafini, Sandra Vincent, Matthew James, Nicolle Domnik, Devin Phillips, Luiz E Nery, Denis E O’Donnell, J Alberto Neder

**Affiliations:** Respiratory Investigation Unit, Division of Respirology, Department of Medicine, Queen’s University, Kingston, ON K7L 2V6, Canada; Respiratory Investigation Unit, Division of Respirology, Department of Medicine, Queen’s University, Kingston, ON K7L 2V6, Canada; Pulmonary Function Tests Laboratory, Federal University of Rio Grande to Sul, Porto Alegre, RS 90040-060, Brazil; Respiratory Investigation Unit, Division of Respirology, Department of Medicine, Queen’s University, Kingston, ON K7L 2V6, Canada; Respiratory Investigation Unit, Division of Respirology, Department of Medicine, Queen’s University, Kingston, ON K7L 2V6, Canada; Respiratory Investigation Unit, Division of Respirology, Department of Medicine, Queen’s University, Kingston, ON K7L 2V6, Canada; Respiratory Investigation Unit, Division of Respirology, Department of Medicine, Queen’s University, Kingston, ON K7L 2V6, Canada; School of Kinesiology and Health Science, Faculty of Health, York University, Toronto, ON M3J 1P3, Canada; Clinical Exercise Physiology Unit, Division of Pulmonology, Department of Medicine, Federal University of Sao Paulo, SP 04021-001, Brazil; Respiratory Investigation Unit, Division of Respirology, Department of Medicine, Queen’s University, Kingston, ON K7L 2V6, Canada; Respiratory Investigation Unit, Division of Respirology, Department of Medicine, Queen’s University, Kingston, ON K7L 2V6, Canada

**Keywords:** artificial intelligence, algorithms, exponential loss, dyspnea, symptoms, COPD

## Abstract

**Objective:**

Heightened muscular effort and breathlessness (dyspnea) are disabling sensory experiences. We sought to improve the current approach of assessing these symptoms only at the maximal effort to new paradigms based on their continuous quantification throughout cardiopulmonary exercise testing (CPET).

**Materials and Methods:**

After establishing sex- and age-adjusted reference centiles (0-10 Borg scale), we developed a novel algorithm (*AI-Techniques Loss-Based Algorithm for Severity Classification* [ATLAS]) based on reciprocal exponential loss for CPET data from patients with chronic obstructive lung disease of varied severity.

**Results:**

Categories of dyspnea intensity by ATLAS—but not dyspnea at peak exercise—correctly discriminated patients in progressively higher resting and exercise impairment (*P < .*05).

**Discussion:**

This new AI-techniques approach will be translated to the care of disabled patients to uncover the seeds and consequences of their activity-related symptoms.

**Conclusions:**

We used innovative informatics research to change paradigms in displaying, quantifying, and analyzing effort-related symptoms in patient populations.

## Introduction

Cardiopulmonary exercise testing (CPET) measures multiple physiological responses (cardiac, respiratory, and muscular) at progressively higher exercise intensities, which are related to their sensory correlates, that is, the intensity of muscular (leg) effort and breathlessness (dyspnea) as quantified by validated scales.^1^^,^^2^ Although these symptoms are reported by the subject at every stage of the exercise test, they are currently analyzed only at peak exercise.^2^ This is a major drawback as less impaired patients may reach higher exercise intensities than the more severe patients, ending up reporting higher symptom scores.^3^ Restricting the analysis to a single discrete score also precludes an appropriate correlation with the concomitant physiological phenomena, a key tenet of CPET to tease out the contribution of the heart, lungs, and muscles to cause exercise intolerance.^4^

**Figure 1.**
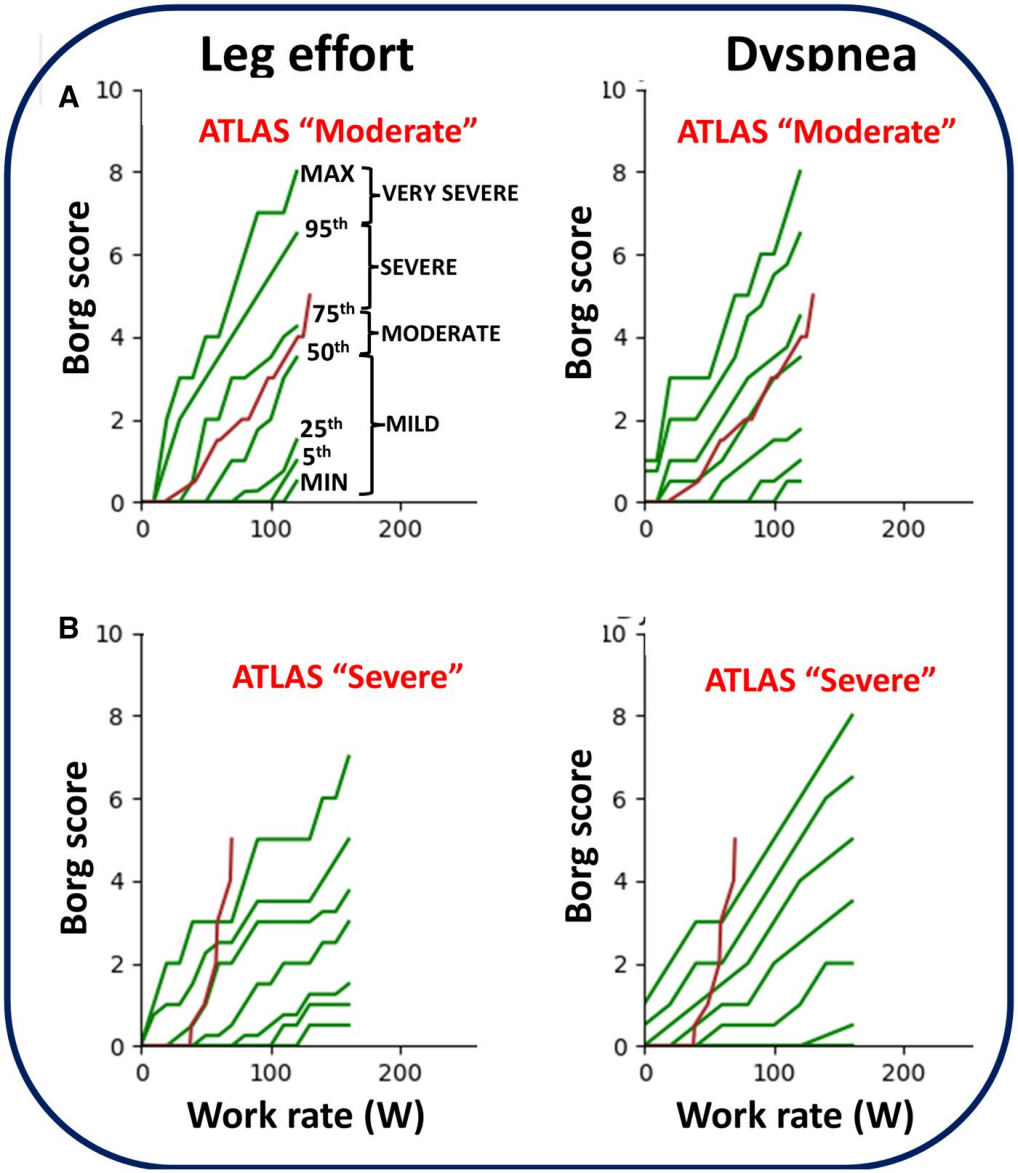
Illustrative examples of ATLAS output in 2 patients with COPD who reported the same peak leg effort and dyspnea scores (5/10 according to the Borg score). Patient #1 (A) is a 58-year-old man with mild-to-moderate COPD-based on resting functional data showing a peak work rate of 115 W. In this case, reported scores of both leg effort and dyspnea remained within the 50th and 75th prediction centiles, ie, “moderate” burden according to ATLAS. Patient #2 (B) is a 50-year-old man with severe COPD based on resting functional data showing a peak work rate of only 50 W. Contrary to patient #1, the reported scores of both leg effort and dyspnea crossed multiple prediction centiles: ATLAS pointed out to “severe” burden despite the same peak scores compared to subject #1.

It is, noteworthy, however, that heightened sensations of leg effort and dyspnea are also reported by unfit but otherwise healthy subjects,^5^^,^^6^ making it difficult to differentiate whether they can be merely a reflection of deconditioning in diseased subjects. In an attempt to circumvent these limitations, the physiological and medical members of our research team have recently developed a comprehensive array of normal reference values for both symptoms *throughout* the exercise test in men and women aged 19-85, that is, minimum and maximal values plus 5th, 25th, 50th, 75th, and 95th centiles (Figure 1).^7^^,^^8^ Thus, we innovated by making the expected symptom burden available in normal sedentary subjects as a given exercise intensity (work rate).

Analyzing these data, however, is fraught with complexities since individual scores of prospective patients were found to cross multiple centiles (Figure 1B), making it difficult to ascertain which centile best described the symptom’s “trajectory,” that is, its cumulative intensity. Moreover, symptoms’ courses were highly variable and, not infrequently, increasing suddenly (Figure 1), followed by periods of stability and vice versa. Thus, the clinical missing gap that prompted our study was: How to best develop an automated algorithm to correctly classify which category (the prediction dyspnea centiles) a particular set of observations (the recorded dyspnea scores in patients) belongs to? The answer to this research question would allow us to establish the basis of novel, user-friendly software for objectively quantifying these disabling symptoms during clinical CPET. To reach a cogent answer to this overarching question, our challenge was to foster a productive interaction between clinical researchers and physiologists with medical informatics specialists who could use innovative computational and mathematical modeling applied to the uniqueness of our patient-reported data.

## Methods

### Algorithm description

#### The overall approach

Our main goal was to establish an algorithm that could be applied to varying sets of data independent of their source. Due to inherent biases when assessing the performance of any model, we opted to use an approach underscored on the mathematical basis of machine learning, as opposed to doing any learning or training; hence AI-Techniques. This implies that the approach should minimize biases, and further improvements could be achieved by enhancing the reference centiles. Recognizing that our fundamental challenge was to correctly classify a given set of serial within-subject observations relative to expected values if the disease was absent (ie, the normal standards for leg effort and dyspnea previously developed by our group),^7^^,^^8^ we used the reciprocal of exponential loss (REL) functions.^9^ We opted for REL given its convex shape, which grows exponentially with an increased deviation, that is, it is exquisitely sensitive to detecting outliers (unexpected lower or higher symptom scores),^10^ allowing a dynamic and continuous weighting that best rewards cases that are consistently near a reference centile. Thus, our algorithm (*AI-Techniques Loss-Based Algorithm for Severity Classification*: [ATLAS]) seeks to identify the inter-centile interval that best describes the observed “trajectory” of the recorded symptom scores (with respect to the published reference centiles)^7^^,^^8^ as the work rate increases with exercise progression (Figure 1). Mathematical details are provided in the Supplementary File S1.

#### The algorithm’s core

To determine the inter-centile range that best represents the recorded symptom from patients, we first calculate each centile’s REL against the CPET data. Since we use a reciprocal, a better fit will result in a greater score, not lower, as used in conventional loss functions. Then, each inter-centile range is given a score that is determined by multiplying the REL of the range’s bordering centiles, for example, the score for the range 25th-50th is the product of the REL of the 25th and 50th centiles. This provides a group score between each reference centile, pinpointing each inter-centile range’s ability to characterize the CPET data. These group scores are normalized such that their sum equals one, providing a percent probability for each range to constitute the most representative centile.

We employ an approach resembling a generalized binary search to further strengthen our confidence that the best inter-centile range has been chosen, even in edge cases where the output is more uniform.^11^ This prevents bias from some target ranges encompassing fewer centile ranges, that is, having a smaller number of values. Thus, we set an initial threshold value to compare the sum of percentages above and below that threshold: the side with the highest value is characterized as the most representative inter-centile range for that set of observations. Several other operations were added to improve further the algorithm’s performance on the CPET data: reweighing, clamping, minimal loss value, and interpolation. They are discussed in detail in the Supplementary File S1.

### Study population

Three hundred fifty-nine sedentary patients (203 men) with chronic obstructive pulmonary disease (COPD)^12^ who had been recruited to take part in ethically-approved investigations comprised the study population. All referred patients safely underwent testing; specifically, no patient was denied assessment based on senescence. The Global Initiative for Chronic Obstructive Lung Disease (GOLD) classification characterized their resting functional impairment vis-à-vis the key physiological variable, that is, the forced expiratory volume in 1 second (FEV_1_).^13^ This sample was highly representative of the COPD patients usually referred to CPET, considering the whole range of disease severity (GOLD stages I-IV). Stepwise 1-2-min staged incremental CPET (10-20 W)^1^^,^^2^ was conducted on an electronically braked cycle ergometer.^14^ Participants rated the intensity of leg effort and dyspnea in the last 30 seconds of each stage and at peak exercise using the 0-10 Borg scale.^15^ The Borg scale is widely used to assess the intensity of effort-related symptoms. It was initially developed to measure the “perceived exercise intensity” and later modified to rate the intensity of breathlessness and peripheral muscle (leg) discomfort. It is considered a category-ratio scale that follows a basic psychophysics rule, which considers that symptoms increase as a power function of the stimuli. The scale ranges from 0 to 10, with “0” being the rest condition (by definition, no effort-related symptom) and “10” the worst shortness of breath ever felt, for example, equivalent to the shortness of breath experienced when the patient was seen in the Emergency Department with a COPD flare. Descriptive short sentences are associated with specific numbers, helping the subject further gauge the symptom intensity relative to the extremes.^15^ Based on pilot studies with ATLAS, the severity of whole-test leg effort and dyspnea was classified as “mild” (minimum-50th centile), “moderate” (51th-75th), “severe” (76th-95th), and “very severe” (>95th). The severity of peak scores was classified as “mild” (0-2), “moderate” (3-4), “severe” (5-6), and “very severe” (≥7).^15^ Also, based on CPET data, subjects were separated into those who showed or did not show critically high lung-mechanical constraints (ie, they were truly limited by “the lungs”)^16^ and those who showed or did not show poor exercise tolerance, that is, peak O_2_ consumption below the lower limit of normal.^17^

### Data analysis

The Statistical Package for the Social Sciences version 21 (SPSS, IBM) was used for all analyses. Differences in patients grouped by GOLD severity were tested using a non-paired t-test. We used McNemar’s test to assess whether there was a significant disagreement between leg effort and dyspnea indicated by ATLAS vs that indicated by the peak scores. Given the inherent absence of an indisputable ground truth relative to individual sensory experiences, we use the closest physiological correlates of symptom severity in COPD to establish which approach (peak scores or ATLAS) better-discriminated patients into progressively worse disease severity: (1) GOLD stage III-IV (severe-to very severe) rather than I-II (resting functional), (2) presence or not of critically high lung-mechanical constraints (exercise functional), and (3) preserved or reduced peak O_2_ consumption (disablement) (chi-squared tests). A *P < .*05 degree of significance was used for all analyses.

## Results

Subjects showed a wide range of disease severity (FEV_1_ ranging from 19% to 108% predicted) with a relatively even biological sex distribution. Based on the GOLD criteria, the resting impairment was considered “mild-moderate” or “severe-to-very severe” in 184 and 175 patients, respectively. Advanced pulmonary function tests confirmed greater functional impairment in GOLD 3-4 patients (*P < .*05) (Table 1).

**Table 1.** Resting characteristics of the COPD sample stratified by GOLD stages.

Variables	**GOLD I-II (Mild-moderate)** **(N = 184)**	**GOLD III-IV (Severe-very severe)** **(N = 175)**
**Demographic/Anthropometric**		
**Men**, *N* (%)	97 (52.7)	100 (57.1)
**Age**, years	66.3 ± 8.9	63.8 ± 9.4^a^
**Body mass index**, kg/m²	28.2 ± 6.3	26.6 ± 5.6^a^
**Lung Function**		
**FVC**, % predicted	95.9 ± 16.2	66.8 ± 15.3^a^
**FEV_1_,** % predicted	71.7 ± 14.5	35.6 ± 9.1^a^
**FEV_1_/FVC**	0.58 ± 0.08	0.42 ± 0.08^a^
**TLC**, % predicted	108.8 ± 14.9	122.7 ± 18.9^a^
**IC**, % predicted	92.3 ± 19.2	65.2 ± 18.2^a^
**FRC**, % predicted	123.5 ± 32.3	175.5 ± 44.0^a^
**RV**, % predicted	141.4 ± 48.6	229.9 ± 75.5^a^
**DL_CO_**, % predicted	69.5 ± 20.6	49.0 ± 17.6^a^

Abbreviations: FVC, forced vital capacity; FEV_1_, forced expiratory volume in 1 s; TLC, total lung capacity; IC, inspiratory capacity; FRC, functional residual capacity; RV, residual volume; DL_CO_, lung diffusing capacity for carbon monoxide.

a *P* < .05.

**Figure 2.**
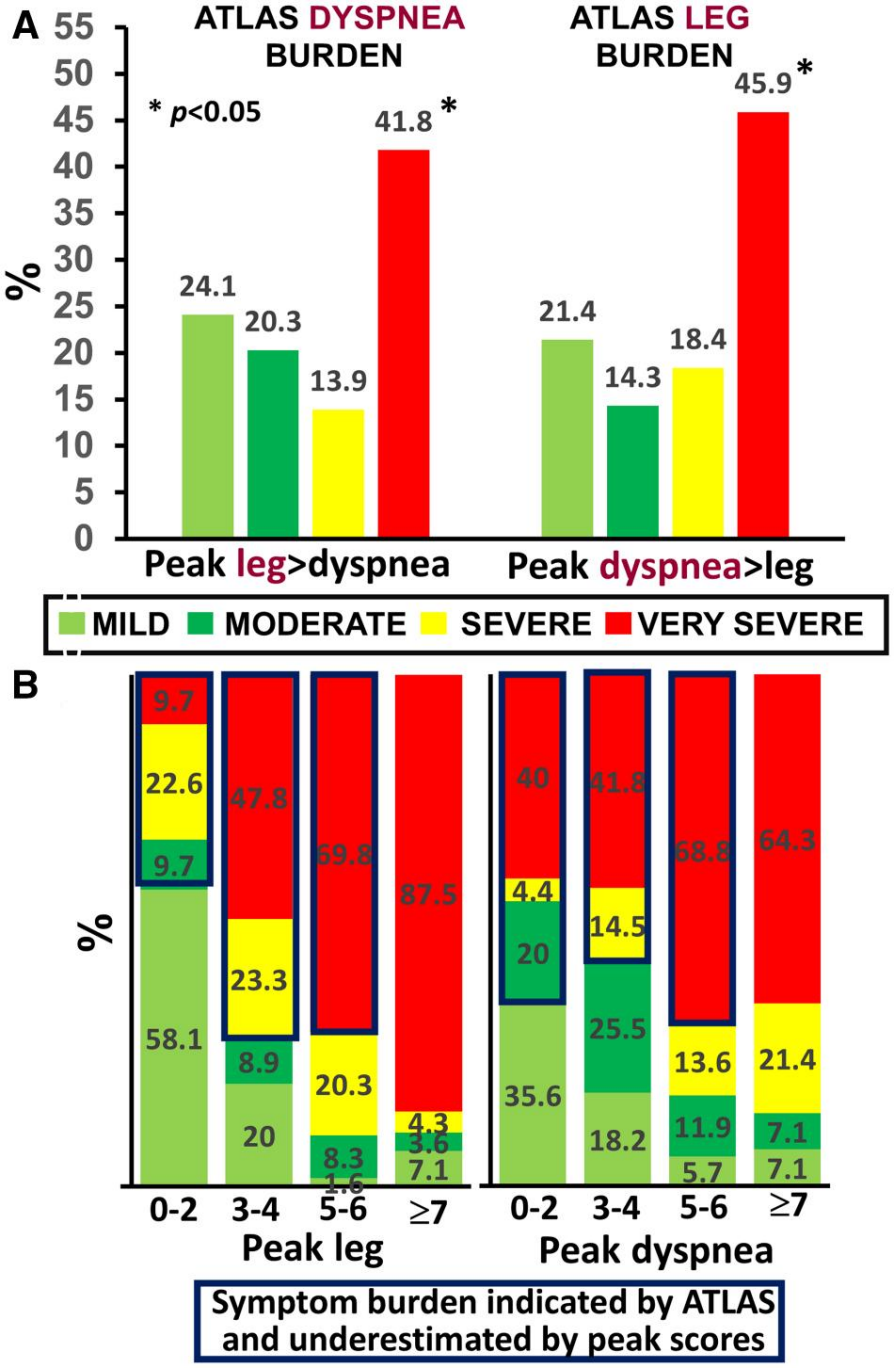
The severity of exertional dyspnea and leg effort as measured by our algorithm throughout the exercise (ATLAS) vs that indicated by the isolated symptom assessment at peak exercise in patients with COPD. A shows that a large fraction of patients in whom leg effort was the dominant symptom had severe-to-very severe dyspnea, according to ATLAS. The opposite was found in those reporting peak dyspnea greater than leg effort. Consistent with these findings, B shows that ATLAS uncovered a larger burden of symptoms compared to peak scores. * *P* < .05 for a significant between-classification disagreement (McNemar’s test). Based on ATLAS, the severity was classified as “mild” (minimum-50th centile), “moderate” (51th-75th), “severe” (76th-95th), and “very severe” (>95th). The severity of peak scores was classified as “mild” (0-2), “moderate” (3-4), “severe” (5-6), and “very severe” (≥7).^15^

**Figure 3.**
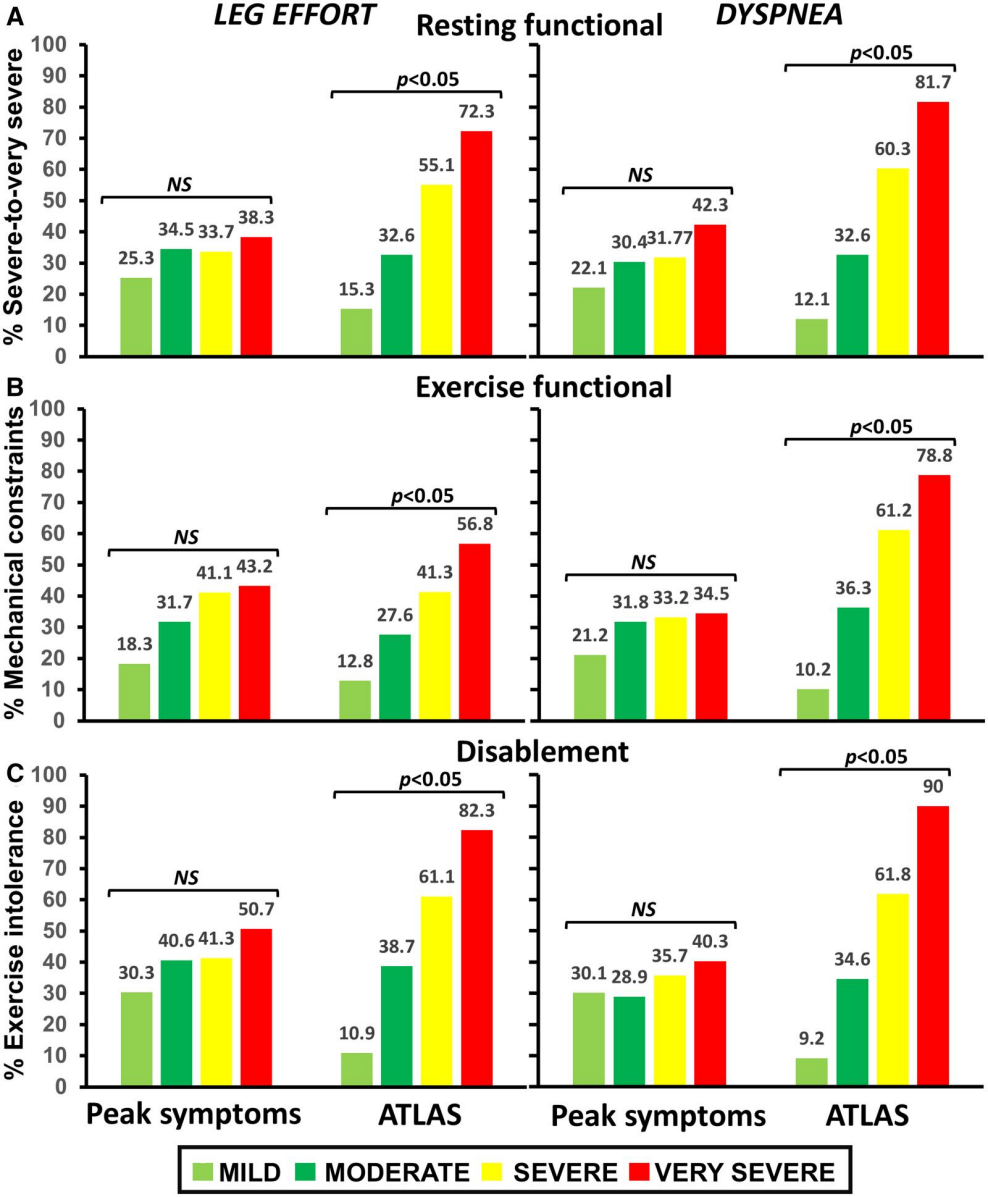
Categories of leg effort (left) and dyspnea (right) severity based on peak scores and ATLAS vs markers of severe resting functional impairment (A), exercise functional impairment (B), and disablement (C) in patients with COPD. Regardless of the metrics, ATLAS categories—but not peak scores categories—consistently discriminated patients at progressively higher levels of impairment and disability. *P < .05 (chi-squared test). Based on ATLAS, the severity of was classified as “mild” (minimum-50th centile), “moderate” (51th-75th), “severe” (76th-95th), and “very severe” (>95th). The severity of peak scores was classified as “mild” (0-2), “moderate” (3-4), “severe” (5-6), and “very severe” (≥7).^15^

The McNemar’s test showed a significant disagreement between the categorical classification of leg effort and dyspnea according to peak scores vs ATLAS *(P <* .05). For instance, leg effort was the main limiting symptom in 158/354 patients (44.6%); 88 of them (55.7%) showed “severe-to-very severe” submaximal dyspnea. Conversely, dyspnea was the main limiting symptom in 99/354 patients (28.0%): 45 of them (64.18%) showed “severe-to-very severe” submaximal leg effort (*P < *.05) (Figure 2A). Moreover, peak leg effort and dyspnea typically underestimated their individual submaximal burden (Figure 2B) (*P <* .05). As shown in Figure 3, the categories of dyspnea severity by ATLAS—but not dyspnea at peak exercise—correctly discriminated patients in progressively higher levels of resting impairment (panel A), exercise impairment (panel B), and disablement severity (panel C) (*P <* .05). Further analysis on ATLAS performance vis-à-vis its ability to discriminate the groups and the degree of (un)certainty at each level of dyspnea severity in both groups is provided in the Appendix S1.

## Discussion

We employed loss functions for classification, a computationally feasible approach familiar to machine learning and mathematical optimization studies,^9^^,^^18^ to identify which category a particular observation belongs to when there are multiple potential outputs in the face of highly variable inputs (Figure 1). Our main findings were: (1) there was a significant disagreement between ATLAS and peak scores in classifying symptom severity in a large sample with varied COPD severity (Figure 2A), with ATLAS better exposing the actual symptoms’ burden (Figure 2B) and (2) ATLAS was superior to peak scores in classifying patients in progressively higher levels of resting and exertional impairment (Figure 3).

Our innovative computational and mathematical modeling approach (ATLAS) has filled a long-lasting clinical need: How can we accurately quantify the burden of exertional symptoms when they change dynamically—and often unexpectedly—as a function of exercise intensity in patients?^3–6^^,^^19^ ATLAS stemmed from a productive interaction between internal medicine, experimental physiology, applied mathematics, and biomedical informatics to answer a clinically relevant question. The interdisciplinary group developed a work plan underscoring the need to make symptom scores available throughout the exercise test, expressing them as a function of the work performed. At this point, medical informatics and mathematical inputs were instrumental, given the non-linear nature of the signal and the absence of “ground truth” for a subjective experience. Several interactions among the team members followed with the intent to develop an algorithm that could properly consider the sizeable inter-subject variability in symptoms under the modulating influences of sex and age. A decision was jointly made to consider gradually higher categories of symptom intensity based on centiles distribution from reference data prospectively obtained in carefully selected normal subjects, ie, rather than a dichotomous “normal” vs “abnormal.” The major challenge to the algorithm development was related to the fact that reported scores frequently crossed multiple centiles, creating uncertainty regarding the best centile to classify individual subjects. Continuous feedback from the clinical team was foremost, guaranteeing that busy clinicians could intuitively understand the algorithm output (numerical and graphical). In this context, it was crucial to ensure that the biomedical informatics solutions were planned to meet future medical expectations concerning the actual program output. Upon successful development of ATLAS, the researchers established a long-term collaborative group to address related CPET issues in which applied mathematics and biomedical informatics were considered sine qua non.

We tested ATLAS’ performance in a population where both heightened leg effort and dyspnea are relevant symptoms,^20^ guaranteeing high external validity for our findings. Thus, this study translated concepts across disciplines, starting with a clinical data source (CPET) in which analysis created knowledge through AI/machine learning-related approaches to develop a novel algorithm that will directly impact clinical decision-making vis-à-vis the interpretation of clinical CPET in patient populations. Of note, ATLAS was significantly more sensitive than the current peak exercise approach in exposing the actual severity of patients’ disablement. This is of particular clinical relevance, allowing the healthcare provider to optimize the required therapeutic and/or rehabilitative interventions for the more impaired subjects. Traditional metrics to assess the accuracy/precision of supervised machine learning models were not employed since the “actual or true” symptom intensity is, naturally, unknown. Nevertheless, ATLAS’ output consistently outperformed the current approach (peak scores) in classifying patients in progressively higher levels of resting and exercise impairment (Figure 3). Thus, the current study included several steps of the pipeline from data generation to clinical application, as proposed by Albers and co-workers.^21^

This study establishes the basis of new paradigms to interpret the submaximal responses to CPET, which can be extended to other subjective experiences (eg, angina, fatigue) and objective (respiratory, cardiac, muscular) adaptations to exercise. Compared to the current reductionist approach of analyzing only a discrete data point at peak exercise, ATLAS allows a continuous assessment of the CPET responses under the rapidly changing conditions that characterize dynamic exercise. Clinically, this is poised to enhance CPET’s diagnostic yield vis-à-vis the mechanisms causing the disabling symptoms of breathlessness and muscular discomfort in patient populations.

Several pre-emptive considerations were discussed among our team members to ensure the development of a novel method that could appropriately capture the severity of rapidly evolving exertional symptoms (Figure 1). By doing so, a large body of data generated during CPET can be medically assimilated and deployed to individualize treatment strategies^22^ to alleviate the burden of disabling exertional symptoms. First and foremost, the algorithm considers the varying widths of inter-centile ranges with respect to the CPET’s data points (Figure 1). For instance, if the CPET’s data point is near a boundary of an inter-centile range, it significantly increases the likelihood of belonging to the neighboring range. Additionally, the algorithm considers instances where the space between many centiles is 0, for example, the minimum, 5th and 25th prediction centile for leg effort up to 80 W in Figure 1A. This fundamental consideration eliminates the ability to analyze individual points’ location between centiles. We also reasoned that it would be essential to recognize that a single-point approximation—like using an average of data points or peak values—falls short due to the significant loss of information (Figure 1).

Loss function classification is a widely adopted method in machine learning designed to quantify discrepancies between 2 vectors with a singular value.^9^ Employing a loss function allows for complete data utilization while eliminating issues with overlapping reference lines. Selecting a suitable loss function (exponential) was crucial, as their behavior varies based on the function and input values.^9^ We carefully defined the algorithm’s leniency, that is, how likely the algorithm is to consider an alternative inter-centile range compared to the best fitting range.^10^ For instance, if a CPET dataset follows just above the 50th centile, the likelihood that the algorithm will consider the data set to belong below the 50th centile is its leniency. We employed several pre-emptive approaches on ATLAS to minimize this risk, such as REL and normalization. Given the superior performance of ATLAS relative to current practice in classifying symptoms burden (Figures 2 and 3), our lenience approach seems adequate for the specific purpose of our algorithm. Using REL to optimize for low lenience proved particularly useful, especially due to the variable widths of individual inter-centile ranges. We recognize that additional investigations are warranted to test ATLAS’ performance when its leniency is changed, either using REL or other loss functions.

### Limitations

While ATLAS may have applications for other related use cases, many additional operations, such as reweighing inertial data points, consider the specific characteristics of CPET’s leg effort and dyspnea data. When generalizing this algorithm to other use cases, these additional computations may hinder its ability to classify the data correctly. Moreover, the specific data point ranges (0-10 scores) and the use of a loss function may need to be modified to alter the lenience or better fit the characteristics of the analyzed data.

## Conclusions

An interdisciplinary group of clinicians, physiologists, applied mathematicians, and biomedical informatics specialists developed the first algorithm (ATLAS) to display, quantify, and analyze leg effort and dyspnea as the exercise intensity increases during incremental CPET. This novel approach is superior to the current practice of assessing these symptoms only at peak exercise, setting the stage for clinically friendly software to amalgamate physiological and sensory data for the care of individual patients.

## Supplementary Material

ocaf051_Supplementary_Data

## Data Availability

The data underlying this article cannot be shared publicly in order to protect the privacy of individuals represented in the dataset.
