# Supplementary material for: AI-Techniques Loss-Based Algorithm for Severity Classification (ATLAS): a novel approach for continuous quantification of exertional symptoms during incremental exercise testing
Source: J Am Med Inform Assoc. 2025 Mar 27;33(1):220–6. doi: 10.1093/jamia/ocaf051 (PMC12758462; doi:10.1093/jamia/ocaf051)
Supplement: ocaf051_Supplementary_Data [file ocaf051_supplementary_data.docx]

**AI-Techniques Loss-Based Algorithm for Severity Classification: (ATLAS): A Novel Approach for Continuous Quantification of Exertional Symptoms during Incremental Exercise Testing**

**Appendix**

**Additional operations**

Beyond the general operations as outlined for this algorithm, we found that there was a need for additional operations to maximise the accuracy of this approach. These were found and implemented specifically for the exertional symptoms we assessed and may need to be modified when employing ATLAS for different purposes.

*Reweighing data points*

Data points were reweighted because individuals often experience an "inertial" increase in reported symptoms as the exercise starts and because the scores may change abruptly just before the exercise termination. The inflection point of this delayed increase can provide insights into the physiological underpinnings of the exertional dyspnea and is essential for determining the severity of an individual's dyspnea. However, these are usually short-lived, and while exponential loss is sensitive to misclassifications, additional weighting is necessary to support the algorithm by applying additional weighting to these short-lived points. For this reason, anytime there is a rapid increase of dyspnea beyond a set threshold, the previous datapoint is set to be equal to the ensuing datapoint, adding more weight to the inertial component.

*Clamping data points*

Given the overarching principle that as the observed data moves further away from a given centile, it simultaneously approached another, we need to establish a maximal range of possible outputs. In other words, if the values are not properly clamped to a maximum and a minimum (derived from the reference values), and the dataset moves beyond the limits of the centiles, there would be a decrease in the probability of all inter-centile ranges without a compensatory increase in another range. Clamping the values to equal the maximum or minimum centiles also maximizes the reciprocal loss, allowing for a significant increase in the respective range.

*Establishing a minimal loss value*

A minimum loss value is required because if clamping the data, to give a centile a perfect loss of 0, an error would occur when taking its reciprocal. Instead, a minimum value of 10^-6^ is employed to adequately represent the perfect fit of the dataset against a centile without causing an error.

*Interpolating the reference values*

The reference values are displayed at specific work rates. Depending on the exercise testing protocol, the available data does not precisely align with the reference values' work rate. In this scenario, the reference values are linearly interpolated considering the two adjacent values.

**ATLAS procedure**

**Preparing the data**

Let P represent the y-axis values of the patient data, at the x-axis values of i, where i is a vector of all x-axis values belonging to the patient data.

$$P\left( i \right)$$

Let R represent the y-axis values of the reference data, where c is a vector representing each centile, and j is a vector of all x-axis values for the reference data.

$$R\left( c,j \right)$$

Note that the values of P and R on the x-axis are different, so an interpolation of R must be created to create indices at all x values of i from j.

**Linearly interpolate reference lines to patient lines:**

R(c,i) represents the interpolated reference lines to go from x values j to i if j ≠ i.

$$R\left( c,i_{n} \right)=R\left( c,a \right)+\frac{R\left( c,b \right)}{R\left( c,a \right)}\cdot\left( i_{n}-a \right)$$

Where a is the first value of j before the n^th^ value of i, and b is the first value of j after the n^th^ value i.

**Clamp the values:**

$$P\left( i_{n} \right)=min(\max\left( P\left( i_{n} \right),R\left( c_{min},i_{n} \right) \right)R\left( c_{max},i_{n} \right))$$

**Reweighing of the points:**

$$v_{1}=\left( i_{n},P\left( i_{n} \right) \right)$$

$$v_{2}=\left( i_{n+1},P\left( i_{n+1} \right) \right)$$

$$v_{1}\cdot v_{2}=i_{n}\cdot i_{n+1}+P\left( i_{n} \right)\cdot P\left( i_{n+1} \right)$$

$${\cos\theta}_{i}=\frac{v_{1}\cdot v_{2}}{\left| v_{1} \right|\cdot\left| v_{2} \right|}$$

$$P\left( i_{n} \right)=P\left( i_{n+1} \right) if {\cos\theta}_{i}\geq\cos80˚$$

Where v_1_ represents the x and y coordinates of P at the n^th^ value of i, v_2_ represents the x and y coordinates of P after the n^th^ value of i, and cosθ_i_ is the angle between P at the n^th^ value of i, and its preceding point. The value of P at the n^th^ value of i is set to be equal to its preceding point if the angle is greater than or equal to 80˚ from the y axis. The 80˚ threshold was selected based on a post hoc analysis involving a random sample showing that the 10˚ of freedom from upright was large enough to be considered inertial without being too lenient. However, as we refine the algorithm further, we plan to investigate what constitutes the best threshold angle systematically.

**Loss formula**

**Exponential loss algorithm:**

$$\mathcal{L}(y-\hat{y})=\sum_{i=0}^{p} \frac{e^{|y_{i}-\hat{y}_{i}|}}{p}$$

**Modified exponential loss to be reciprocal with minimum loss for each centile with a minimum loss value:**

$$L\left( c_{m} \right)\mathcal{=L}(P\left( i \right)-R\left( c_{m},i \right))=\sum_{n=0}^{p} \frac{p}{max\left( e^{|P\left( i_{n} \right)-R\left( c_{m}{,i}_{n} \right)|},{10}^{-6} \right)}$$

Where L(c_m_) is the loss of the patient’s data compared to the m^th^ reference line.

**Getting vector of group scores:**

$${G'}_{k}=L\left( c_{m} \right)\cdot L\left( c_{m+1} \right)$$

Where G’ represents the product of adjacent losses for each centile, such that k = [0, |i|-1], where |i| is the total number of elements within i. G’_k_ now represents the group score at each interval procedurally.

**Normalize:**

$$G_{k}=\frac{{G'}_{k}}{\sum G'}$$

Where G_k_ represents the group scores, normalized to all group scores, at intervals of k.

**Binary classification**

First a threshold is set at a centile, and the sums of group scores above and below this centile are compared. This process is then repeated recursively for the next threshold using only the group scores within the greater set of the first threshold.

The first threshold equals centile c_q_ where q represents the value of m corresponding to the first threshold.

The adjacent group scores are G_u_ and G_v_ such that

$$G_{u}\approx L\left( c_{q-1} \right)\cdot L\left( c_{q} \right)$$

$$G_{v}\approx L\left( c_{q} \right)\cdot L\left( c_{q+1} \right)$$

**A comparison is made:**

$$\sum_{s=0}^{u} G_{s}?\sum_{t=v}^{k} G_{t}$$

If the former is greater, then the process is repeated, by only using G scores with values k between 0 and u, with a new threshold q that lies between 0 and the former value of q. If the latter is greater, then the same process is completed except values of k are between v and |i|-1.

This is repeated until sufficient granularity has been reached, and a range of centiles is determined to have the highest score.

**Quantifying the Binary classification**

Despite the quantification of inter-centile scores, the requirement for a binary classification poses challenges to quantifying ATLAS’ certainty for a given severity. To solve this, we employed a numerical approach. A value of negative or positive two is assigned based on the outcome of the first threshold, being mild/moderate or severe/very severe respectively. Then, this value is multiplied by the relative scoring of mild to moderate or very severe to severe. This generates a scoring system such that values closest to ±2 reflects a strong confidence in mild or very severe, while values closest to 0 reflect a confidence in severe or moderate (depending on whether the value is positive or negative respectively).


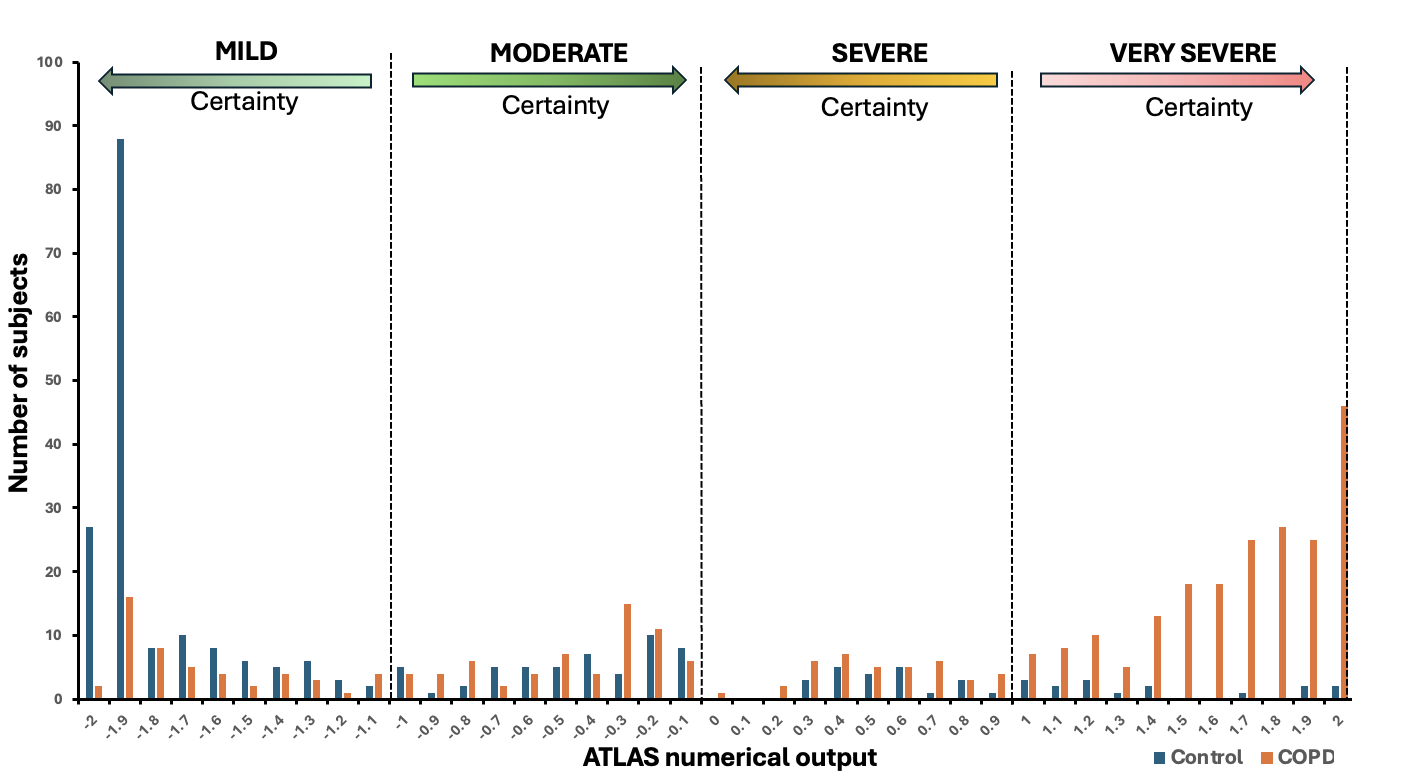


**Figure 1. Histogram of quantified ATLAS outputs on controls (n=275) and COPD patients (n=354) using dyspnea as a function of work rate. Thresholds for mild moderate, severe, and very severe are shown with vertical lines, as well as the directionality of confidence.**

This approach highlights two important factors. The first being that ATLAS has a strong discriminatory power between healthy subjects and COPD patients using just work rate. Secondly, ATLAS tends to have a high confidence when comparing these two grouping to one another.
